# In Vivo Anti-Inflammatory Activity of Lipids Extracted from the Most Abundant Cyanobacterial Strains of the Therapeutic Euganean Thermal Muds

**DOI:** 10.3390/biom15091301

**Published:** 2025-09-10

**Authors:** Micol Caichiolo, Giuliana d’Ippolito, Angela Grazioso, Chiara Rampazzo, Angelica Marchetto, Fabrizio Caldara, Luisa Dalla Valle, Nicoletta La Rocca

**Affiliations:** 1 Department of Biology, University of Padova, Via U. Bassi 58/b, 35131 Padova, Italy; micol.caichiolo@phd.unipd.it (M.C.); chiara.rampazzo.1@unipd.it (C.R.); marchetto.angelica.1998@gmail.com (A.M.); 2 Pietro D’Abano Thermal Studies Center, Via Jappelli 5, 35031 Padova, Italy; fabrizio.caldara@centrostuditermali.org; 3 Institute of Biomolecular Chemistry, Centro Nazionale delle Ricerche, Via Campi Flegrei 34, 80078 Naples, Italy; giuliana.dippolito@cnr.it (G.d.); angela.grazioso@unina.it (A.G.)

**Keywords:** thermal mud, cyanobacteria, lipid extracts, glycoglycerolipids, zebrafish, anti-inflammatory activity

## Abstract

Cyanobacteria are a natural source of bioactive compounds increasingly recognized for their anti-inflammatory properties. In the Euganean Thermal District (Italy), thermal muds, used to cure arthro-rheumatic diseases, are prepared using natural clay and thermal water, resulting in a mature mud characterized by a complex microbial biofilm dominated by Cyanobacteria. Among these, *Phormidium* sp. ETS-05 has been shown to contribute to the therapeutic properties of the mud, mainly through the production of bioactive compounds such as exopolysaccharides (EPSs) and glycoglycerolipids (GLs). In contrast, the role of biomolecules from *Thermospirulina andreolii* ETS-09 and *Kovacikia euganea* ETS-13, also abundant in mature muds but at higher maturation temperatures, has not been investigated. This study focuses on the lipid profiles of these cyanobacteria, cultivated under temperature conditions that mimic their natural environment and that are different for the three species. Lipid extracts were analyzed for GLs classes and fatty acid composition, and their anti-inflammatory potential was assessed in vivo using a zebrafish inflammation model. All extracts showed anti-inflammatory activity with *Phormidium* sp. ETS-05 displaying the highest lipid content and the most rapid and potent beneficial effect, likely due to the specific composition of its GLs, presenting the greatest abundance of polyunsaturated fatty acids. These findings provide new insights into the biological basis of the therapeutic effects of Euganean muds and emphasize the importance of maturation conditions for cyanobacterial growth and bioactive lipid production.

## 1. Introduction

Cyanobacteria are considered a natural sources of bioactive molecules and, being rich in primary and secondary metabolites, they have been used for several applications, including pharmaceuticals ones. Indeed, a wide range of cyanobacterial compounds, including proteins, lipids and polysaccharides, among others, were proved to possess beneficial properties (reviewed in [1]).

Therefore, Cyanobacteria are increasingly recognized for their beneficial effects in mud therapy, also thanks to the biologically active substances that they produce and release during the process of mud maturation [2,3].

The Euganean Thermal District (Padova, Italy) is home to numerous spas and thermal centers offering a wide range of therapeutic treatments, and it is especially renowned for the use of therapeutic muds, also called peloids, which have been recognized by the Italian Health System for their effectiveness in alleviating pain associated with arthro-rheumatic diseases [4,5]. The distinctiveness of these muds derives from their maturation process, carried out independently by each spa in the Euganean area following specific rules that allow to obtain the “Mature Mud AOC” certification [6]. Briefly, mud collected from local thermal lakes is placed in open ponds or tanks and covered with flowing thermal water for no less than two months, fostering the growth of a complex microbiota. An in-depth analysis of mud maturation parameters and the associated microbial composition across numerous spas of the Euganean District revealed the presence of a microbial community mainly composed of Cyanobacteria, Chloroflexi, Bacteroidetes and Proteobacteria. Water temperature was recognized as the main environmental factor promoting changes in Cyanobacteria total abundance and species composition. In particular, at a maturation temperature ranging between 37 and 47 °C (that is the suggested mud maturation range), a stable microbial community, with the highest cyanobacterial abundance and *Phormidium* sp. ETS-05 as the main species, is achieved [7]. Conversely, at higher maturation temperatures, up to 55 °C, a complete modification in the cyanobacterial composition was detected, with a significant increase in the abundance of the species *Thermospirulina andreolii* ETS-09 and *Kovacikia euganea* ETS-13 [7,8].

*Phormidium* sp. ETS-05 has already been shown to produce different bioactive molecules that could contribute to the therapeutic effects of mud applications. Indeed, its lipidic and polysaccharidic compounds possess validated anti-inflammatory and immune-modulatory properties, both in vitro and in vivo [9,10,11,12]. With regard to lipidic compounds, glycoglycerolipids (GLs) represent a class of primary metabolites of growing interest due to their recognition also as antibacterial, antiviral and anti-tumoral molecules, besides anti-inflammatory activities. GLs are normally classified into three wide classes according to the features of their glycosidic head: monogalatosyldiacylglycerols (MGDGs), digalactosyldiacylglycerols (DGDGs) and sulfoquinovosyldiacylglycerols (SQDGs). Together with phosphatidylglycerol (PG), they are the main components of cytoplasmic and thylakoid membranes of Cyanobacteria [13,14]. So far, to evaluate the contribution of these molecules to the therapeutic effectiveness of Euganean peloids, GLs have been extracted and biochemically characterized just from *Phormidium* sp. ETS-05, cultivated at 30 °C, a temperature lower than that of the suggested mud maturation range [15]. The different GLs classes were tested in vitro and in vivo and showed antioxidant and anti-inflammatory capabilities [9,10,11]. These findings led to obtaining an “European Patent” in 2013 [16] and for this reason, *Phormidium* sp. ETS-05 is recognized today as the target species of mud maturation.

Recently, the cyanobacterial strains *Thermospirulina andreolii* ETS-09 and *Kovacikia euganea* ETS-13, that are particularly abundant in mud matured at higher temperatures (above 45 °C), have been isolated and underwent polyphasic characterization [17,18].

These species have never been studied for their contribution to the beneficial properties of the final product. Even if some data are available for the properties of ETS-05 GLs, no analyses were performed on lipids of the newly isolated species *Phormidium* sp. In addition, since variations in temperature have been shown to affect growth as well as lipid production and quality [19,20], the previously obtained results on *Phormidium* sp. ETS-05 lipids, cultivated at 30 °C, should be updated by considering its optimal growth temperature, recently identified to be 40 °C by Zampieri et al. [21].

In this study, the most abundant cyanobacterial species found in the mature muds of the Euganean Thermal District (*Phormidium* sp. ETS-05, *Thermospirulina andreolii* ETS-09 and *Kovacikia euganea* ETS-13, hereafter named only with their ETS code, where ETS stands for Euganean Thermal Strain) were cultivated under their optimal growth temperatures. These conditions were selected based on previous works performed with ETS-05 and ETS-13 [18,21] and preliminary experiments with ETS-09. Moreover, the adopted temperatures reproduce those in which these organisms have been found as predominant in mature muds, as determined through NGS analyses of environmental samples [7,8]. Total lipid amount, content in GLs classes and fatty acids composition were subsequently analyzed for the different cyanobacteria cultures. Finally, to unravel the potential contribution of each species in the therapeutic properties of the peloids, the anti-inflammatory potential of their total crude lipid extracts was tested in zebrafish, a model organism widely adopted for in vivo drug screening [22,23,24].

## 2. Materials and Methods

### 2.1. Cyanobacterial Strains and Maintenance

Cultures of ETS-05, ETS-09 and ETS-13 were cultivated in the laboratory thanks to a previous collaboration with the Pietro d’Abano Thermal Studies Center [17,18,25]. Organisms were kept in flasks in a thermostatic chamber at 30 °C (±1 °C) and 30 μmol photons m^−2^ s^−1^ continuous white light. BG11 medium was used for maintenance and growth of ETS-05 and ETS-13 strains [26], while BG11 with HEPES (10 mM, pH 8) was used for ETS-09 strain.

### 2.2. Cyanobacterial Strains Cultivation at Optimal Growth Conditions

ETS-05 and ETS-09 organisms were cultivated using the Photobioreactor Multi-cultivator MC-1000 OD system (Photon Systems Instruments, Drásov, Czech Republic). The different cyanobacteria were pre-acclimated to the growth in photobioreactors using cultures renewed with fresh media. In particular, ETS-05 was pre-acclimated for 4 days at 40 °C and 50 μmol photons m^−2^ s^−1^ starting from an optical density at 750 nm (OD_750nm_) of 0.4 while ETS-09 was pre-acclimated for 4 days at 45 °C and 50 μmol photons m^−2^ s^−1^ starting from an OD_750nm_ of 0.3. Light was provided in continuos by cold white LEDs. A constant flux of filtered atmospheric air bubbles was used to maintain the filament in suspension within the medium. Subsequently, cells were decanted to eliminate residues of released compounds present in the medium. Inocula for the experiments were obtained by resuspending these pre-acclimated cultures to an OD_750nm_ of 0.2 in a final volume of 80 mL for each tube. ETS-05 growth was then conducted at 75 μmol photons m^−2^ s^−1^ at 40 °C for 5 days (according to [21]). Nine biological replicates were carried out.

ETS-09 growth was also performed at 75 μmol photons m^−2^ s^−1^ but at 45 °C for 6 days. Five biological replicates were performed.

The ETS-13 organism was instead grown in flasks placed inside a thermostatic incubator because it tends to produce thick biofilms that hinder sampling and make the growth in stirred or aerated conditions, such as in photobioreactors, unfeasible. The illumination systems used consisted of white light from LED strips. The light spectrum, measured using LI-COR LI-180 spectrometer (Ecosearch Srl, Perugia, Italy), was the same as the white LEDs of the Multi-cultivator. Cultures were pre-acclimated for 7 days starting from about 0.8 g of fresh weight biofilm per 50 mL of BG11 medium at 40 °C and 15 μmol photons m^−2^ s^−1^ of constant light. Cells were then centrifuged to eliminate residues of released compounds present in the medium. Inocula for the experiments, corresponding to about 28 g_DRYWEIGHT_, were obtained by resuspending the cultures with a final volume of 50 mL for each flask. The growth was performed at 15 μmol photons m^−2^ s^−1^ at 50 °C for 14 days (according to [18]). Six biological replicates were carried out.

### 2.3. Growth and Biomass Assessment

OD_750nm_ was measured using a spectrophotometer (Cary100 UV-VIS, Agilent, Milan, Italy) at the starting and end point of the experiments. Specifically, due to the inhomogeneity of the cultures, two samples were taken from each culture, and the OD value was obtained from the media of five measurements on each. Biomass was also evaluated as dry weight of 5 mL of culture using 0.45 μm filters at the starting and end point of the experiments. OD_750nm_ was not measured for ETS-13, and biomass was assessed only as wet and dry weight, due to the formation of thick aggregations. For wet weight measures, samples were centrifuged to remove excess medium and weighted in pre-weighted tubes. For dry weight measures, samples were placed for 24 h at 60 °C until total evaporation and weighted in pre-weighted tubes. The stability of the samples’ weight was also checked after 48 h. The dry weight/fresh weight ratio was used for the calculation of the dry weight per liter of culture (gDW/L).

### 2.4. Chlorophyll a Extraction and Quantification

For the extraction of lipophilic pigments, 1 mL of cell culture for each experimental biological replicate was sampled at the first and last day of growth and centrifuged at 20,000× *g*. The supernatant was discharged, and the pellet was resuspended in 1 mL *N, N*-dimethylformamide (227056, Merk Life Science, Milan, Italy). Each sample was then incubated at 4 °C for 24 h in the dark. Supernatant obtained from centrifugation was then analyzed spectrophotometrically (Cary100 UV-VIS, Agilent, Milan, Italy), recording the absorption spectrum from 350 to 750 nm. Chlorophyll *a* concentration was calculated using the extract absorption value at 664 nm according to the method and equation reported by Moran [27]. Quantities are reported as µg/mL of culture, as a proxy of its biomass concentration.

### 2.5. Lipid Extraction and Quantification

The obtained cyanobacterial biomass was lyophilized in a freeze drier, and 50 mg of the lyophilized pellet was weighed into a glass screw-cap tube. Total lipid extraction was performed according to the Bligh and Dyer method [28] with some adjustments. Briefly, for the initial extraction, 2 mL of chloroform (00802A, Carlo Erba Reagents, Milan, Italy) and 4 mL of methanol (34860, Merk Life Science, Milan, Italy) were added, and the mixture was sonicated for 5 min in an ice-water bath. Following this, 1.6 mL of water (34877, Merk Life Science, Milan, Italy) was added and sonicated for another 5 min. The mixture was then centrifuged at 3000× *g* for 5 min at 4 °C, and the organic phase was carefully collected in a separate tube. For the second extraction, 3.8 mL of a chloroform–methanol–water (1:2:0.8) mixture was added to the remaining pellet, sonicated for 5 min in an ice-water bath and centrifuged at 3000× *g* for 5 min at 4 °C. This organic phase was combined with the previously collected phase and centrifuged again if necessary to remove any suspended particles. To enhance phase separation, 3 mL of chloroform was added to the combined organic phases, vortexed, agitated and allowed to escape air. The mixture was allowed to separate and centrifuged at 1000× *g* for 5 min at 4 °C. The aqueous phase was discarded and the underlying organic phase containing the lipids was retained. The organic phase was evaporated to dryness under nitrogen. The dried lipids were transferred into a pre-weighed vial using a chloroform–methanol (1:2) mixture, then dried again and weighed to determine the total lipid content. The lipid extract was stored in a freezer until further analysis. For the in vitro and in vivo tests, it was dissolved in ethanol at different concentrations, as described in the following paragraphs.

### 2.6. Total Lipid Extracts Chemical Characterization

NMR analysis of lipid extracts from the three cyanobacterial strains was performed with ERETIC method, following the protocols previously described [29,30]. In particular, ^1^H-NMR spectra were acquired on Bruker Avance III 600-MHz spectrometer equipped with an inverse CryoProbe (Bruker BioSpin GmbH, Rheinstetten, Germany). Peak integration, ERETIC measurements and spectrum calibration were conducted with the specific subroutines in the Bruker Top-Spin 3.1 software. The spectra were collected with a spectral width of 14 ppm (8417.5 Hz), incorporating 32 K time-domain data points, a 90° pulse, spectrum size of 32 K, with processing that included 0.6 Hz line broadening for the exponential decay function.

Total lipid extracts were solubilized in 700 μL of a 1:1 (v/v) mixture of CDCl_3_ and CD_3_OD 1:1 and then transferred to a 5 mm NMR tube for ^1^H-NMR analysis (Figure S6). The signal of the doublet at δ 6.90 of 4,4′-dihydroxybenzophenone (DHBP) was used for calibration (2.23 μmol in 700 μL of CDCl_3_/CD_3_OD 1:1). The integration of the following diagnostic signals was used for quantitative analysis of main glycoglycerolipids (GLs): doublet at 4.90 ppm (J = 3.5 Hz) corresponding to the anomeric proton of galactose in digalactosyldiacylglycerols (DGDG), doublet at 4.80 ppm (J = 3.7 Hz) due to the anomeric proton of sulfoquinovose in sulfoquinovosyldiacylglycerols (SQDG) and doublet at 3.88 ppm (J = 3.0 Hz) due to the methine proton at C4 of galactose of monogalactosyldiacylglycerols (MGDG) [30]. The mole number of each glycoglycerolipid class was corrected for the standard recovery and normalized for mg of lipid extract for each technical replicate. Results were expressed as % according to the following formula:

$$\%\;glycoglycerolipid\;class\;=\;\left(\frac{nmol\;of\;glycoglycerolipid\;class}{Sum\;nmol\;of\;all\;glycoglycerolipids}\right)\;\times 100$$

The total fatty acid composition was assessed by GCMS on the corresponding fatty acid methyl esters (FAME), as previously defined [29,31]. FAME were obtained by saponification of the lipid extracts using methanol in sodium carbonate maintaining the reaction at 40 °C [29,30]. After 4 h, the reaction mixture was diluted with milli-Q water to fully dissolve sodium carbonate, neutralized using solution 1 M HCl and extracted three times with dichloromethane. The combined organic extracts were dried using a nitrogen stream, re-dissolved in MeOH to achieve a final concentration of 1 μg/μL and analyzed using GSMS on a Thermo Focus GC Polaris Q instrument with a 5% diphenyl column. The GCMS settings included 70 eV for the ion trap, an injector temperature of 210 °C and a transfer line temperature of 280 °C.

FAME elution was conducted based on the following temperature gradient: starting at 160 °C for 3 min, followed by an initial rise of 3 °C/min until reaching 260 °C, and then a secondary increase of 30 °C/min until attaining 310 °C. Lastly, the temperature was maintained at 310 °C for 7 min. FAME were identified by comparing their retention times and mass spectra with those of standard mixtures (Figure S5). Fatty acid (FA) content of each chemical species was expressed as % of total fatty acids, according to the formula as follows:

$$\%\;FA\;=\;\left(\frac{area\;of\;the\;single\;FA}{sum\;areas\;of\;total\;FAs}\right)\;\times 100$$

### 2.7. In Vitro Cell Viability Assay

BJ skin fibroblasts (CRL-2522™, ATCC, Manassas, VA, USA) were routinely tested for mycoplasma contamination (11-1025, VenorGeMClassic, Minerva Biolabs GmbH, Berlin, Deutschland) and cultured in DMEM with 4.5 g/L glucose (31966-021, Thermo Fisher Scientific, Milan, Italy) supplemented with 10% fetal bovine serum (FBS) (10270106, Thermo Fisher Scientific, Milan, Italy), non-essential amino acids and antibiotics. Cell cultures were maintained at 37 °C in a humidified atmosphere with 5% of CO_2_.

To evaluate the possible cytotoxic effect of the total crude lipid extract from the cyanobacterial strains, cell viability was measured using the Cell Counting Kit-8 colorimetric assay (CCK-8) (B34304, Bimake, Houston, TX, USA). Briefly, cells were seeded in 96-well plates at an initial density of 6 × 10^3^/well and allowed to adhere for 24 h. Cells were treated for 24 and 48 h with increasing concentrations of lipids (from 2.5 to 40 μg/mL), previously dissolved in ethanol. Control groups included untreated cells (CTRL), cells exposed to Triton X-100 (Et-OH) (T8787, Merk Life Science, Milan, Italy) at 1 X concentration (CTRL and Triton X 1X) and cells treated with 0.1% Et-OH to account for solvent effects. After treatment, cells were washed using PBS and replaced with 100 μL of fresh DMEM medium. Subsequently, 10 μL of CCK-8 solution were added to each well and incubated for 2 h at 37 °C. Absorbance was measured using a microplate reader (Tecan Spark^®^, Milan, Italy) at a wavelength of 450 nm, and the absorbance in the control group was considered as 100% cell viability. Cell viability was calculated relative to the untreated controls, considered as 100%, using the formula as follows:

$$Cell\;viability\;=\;\left(\frac{mean\;OD\;of\;treated\;cells\;-background\;absorbance}{mean\;OD\;of\;untreated\;cells\;-background\;absorbance}\right)\;\times 100$$

where OD refers to optical density and untreated cells correspond to CTRL. Data are means ± standard deviation of three biological replicates each analyzed in triplicate.

### 2.8. Zebrafish Maintenance

For all experiments, we used Wild Type (WT) larvae before the free-feeding stage and thus, they did not fall under animal experimentation laws according to EU Animal Protection Directive 2010/63/EU. Zebrafish were reared, bred and staged according to established guidelines [32]. Embryos and larvae were maintained in fish water in Petri dishes at 28 °C. Embryonic and larval stages were reported as hours or days post-fertilization (hpf, dpf). Larvae were anesthetized or euthanized with fish water containing 0.16 or 0.30 mg/mL of tricaine (MS222; E10521, Merk Life Science, Milan, Italy), respectively.

### 2.9. Fish Embryo Acute Toxicity (FET) Test

The fish embryo acute toxicity test (FET) was performed following the indication of the OECD Guideline No. 236 (2013), published by the Organization for Economic Cooperation and Development (OECD, Paris, France) [33]. This test was used to analyze the acute toxicity of the total crude lipid extract of *Phormidium* sp. ETS-05. We adopted the protocol previously described [12]. In addition, since lipids were dissolved in Et-OH, a solvent control was also necessary (0.1% Et-OH). A scheme of the plates’ organization is reported in Figure S1.

### 2.10. Chemical Treatment with Copper Sulphate and Anti-Inflammatory Tests

Inflammation induction was performed on 3-dpf larvae with a copper sulphate solution (CuSO_4_·5H_2_O, 1027841000, Merk Life Sciences, Milan, Italy) prepared in fish water (FW) before the beginning of each experiment. After two hours of treatment with 20 μM copper sulphate solution, samples were washed four times with FW to ensure the removal of any residual compound. After the inflammation induction, lipids dissolved in Et-OH at 2.5, 5 and 10 μg/mL concentration (Figure S2A) were added to FW for different time intervals according to the specific experiment (waterborne exposure procedure). Each experiment was performed three times with 15–20 larvae per replica, and the lipids from the different cyanobacterial strains were tested separately.

#### 2.10.1. Analysis of Morphological Traits and Image Processing

Larval morphology was analyzed after inflammation induction and treatment with total crude lipid extracts for 24 and 48 h, considering the following traits: operculum bone ossification and swim bladder insufflation. Specifically, the experiments were divided into three parallel sets, and, within each set, we compared the effectiveness of total crude lipid extract from the different cyanobacterial strains in promoting recovery from the inflammation-induced developmental delay. For the morphological analysis, larvae were exposed for 24–48 h to the different lipid concentrations, as reported in Figure S2B. The morphological traits analyzed are graphically represented in Figure S3. Alizarin red S staining was adopted to visualize the operculum bone size, which was then measured according to Tarasco et al. [34]. The swim bladder area was normalized for the body length and the operculum area for the head area. After anesthesia with tricaine, larvae were arranged sideways in 2.5% methyl cellulose in microscope slides. Digital micrographs were taken with a Leica M165 FC stereoscopic microscope (Leica Microsystems, Milan, Italy) equipped with a Leica DFC7000 T digital camera (Leica Microsystems, Milan, Italy). Micrographs were analyzed with ImageJ software v1.52a to compare treated larvae and controls.

#### 2.10.2. Locomotor Activity Test

Larval behavior and movement were analyzed following inflammation induction and the subsequent treatment with the lipids extracted from the three cyanobacterial strains. Specifically, larvae were treated for 48 h with 10 μg/mL lipid extract after the exposition to copper sulphate, as reported in Figure S2B. Larvae subjected to inflammatory stress were either exposed to lipids for 48 h or kept in FW, serving as the inflamed control group. Each larva was individually placed in a well of a 48-well plate containing 0.9 mL of FW. The locomotor activity of each larva was monitored using the DanioVision Observation Chamber (Noldus, Wageningen, The Netherlands), with the temperature maintained at 28 °C throughout the experiment. Prior to the test, larvae were acclimatized for 10 min in darkness, followed by three alternating cycles of light and dark, each lasting 10 min. Larval movement was tracked using the EthoVision XT 8.5.614 video tracking software (Noldus, Wageningen, The Netherlands), with distance traveled recorded every 2 min. The overall distance covered by each larva was calculated by summing the distances moved over the entire period of observation.

### 2.11. Statistical Analysis

For statistical analysis we used the Graph Pad Prism (Version 10.2.3, GraphPad software). The statistical analysis of comparison between control and treated samples was performed with one-way ANOVA followed by Tukey’s multiple comparison test or Brown–Forsythe and Welch ANOVA tests followed by Dunnett’s multiple comparison test with individual variances computed for each comparison. Data are presented as means ± standard deviation, unless otherwise specified. The *p*-values are indicated with letters that show the results of the multiple comparison test in all figures, unless otherwise specified.

## 3. Results and Discussion

### 3.1. Cyanobacteria Culture Features

In this study, to obtain the biomass for the following lipid extraction, biochemical characterization and anti-inflammatory activity assessments, the different cyanobacterial strains have been cultivated based on recent studies describing their optimal growing conditions [18,21]. ETS-05 and ETS-09 were grown at 75 μmol photons m^−2^ s^−1^ and at 40 °C and 45 °C, respectively. ETS-13 was instead cultivated at 15 μmol photons m^−2^ s^−1^ and at 50 °C. In particular, the used growth temperatures were compatible with those recorded assessing the distribution of the main different species in the Euganean Thermal District environments [7,8], making the choice even more appropriate for correctly evaluating the composition and bioactivity of the lipid extracts.

To check the status of the strains, referred to as ETS-05, ETS-09 and ETS-13, different physiological and biochemical parameters were analyzed at the beginning and at the end of the culture preparation (OD_750nm_, dry weight-DW and Chl *a*). These results are in line with the growth obtained by Zampieri and collaborators [18,21]. In particular, the mean biomass accumulation for ETS-05 was 0.42 ± 0.09 g/L, starting from 0.23 ± 0.07 g/L, whereas for ETS-09, the DW was 0.54 ± 0.03 g/L on the last day of the growth, starting from 0.35 ± 0.07 g/L. The OD_750nm_ measurements resembled the increase in DW, confirming an active growth of the cultures (0.23 ± 0.06 to 0.51 ± 0.13 for ETS-05 and 0.19 ± 0.05 to 0.75 ± 0.12 for ETS-09). Chl *a* was also augmented accordingly (2.79 ± 0.45 to 6.70 ± 1.69 μg/mL for ETS-05 and 1.20 ± 0.13 to 5.18 ± 0.87 for ETS-09). Regarding ETS-13, a very slow-growing organism that forms aggregations and thick biofilms, the growth assessment was very difficult. Indeed, the biomass content, registered after the two weeks of acclimation, was 0.44 ± 0.16 gDW/L compared to the 0.43 ± 0.13 gDW/L at the beginning of the experiment (considering that the dry weight was tentatively determined based on the ratio between fresh and dry weight). In addition, differently from ETS-05 and ETS-09, an increase in pigment concentration over ml culture was undetectable for ETS-13. However, ETS-13 cultures still showed a green coloration and comparable levels of chlorophyll fluorescence, indicating their good status (Figure S4). Although ETS-13 showed very slow growth and might be thought to be not well acclimated, Hirayama and Kishida [35] demonstrated that, growing *Mastigocladus laminosus*, a phylogenetically close species, at 48 °C and then transferring it to 40 and 55 °C for 4 days, the composition of fatty acid and lipid classed was affected by the growth–temperature shift. So, overall, the growth periods used were considered sufficient to achieve the acclimation of the strains to their specific optimal temperature.

### 3.2. Lipid Extracts Characterization

The obtained biomasses were lyophilized and subjected to total lipid extraction using the Bligh and Dyer method [28], a protocol shown by Sheng et al. [36] to be appropriate for extracting GLs components from cyanobacteria.

**Figure 1:**
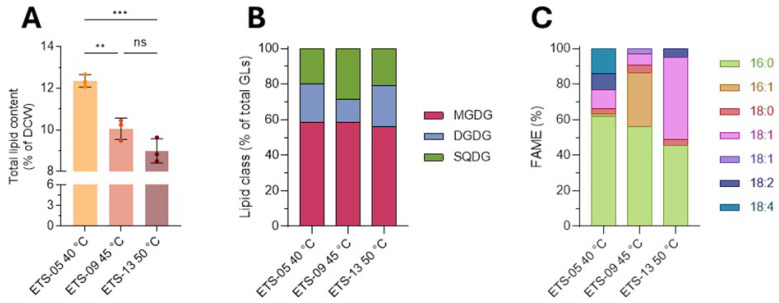
Lipid production and content in ETS-05, ETS-09 and ETS-13. (**A**) Total lipid content expressed as % of Dry Cell Weight (DCW). Statistical analysis was performed using GraphPad Prism 10 (ordinary one-way ANOVA test followed by Tukey’s multiple comparisons test with a single pooled variance), *p*-value: <0.01 **; <0.001 ***; (**B**) Glycerolipid composition, expressed as % over the total GLs amount (reported in Table S2 as nmol/mg lipid extract). MGDG, monogalactosyldiacylglycerols; DGDG, digalactosyldiacylglycerols; SQDG, sulfoquinovosyldiacylglycerols; (**C**) fatty acid composition (FAME) expressed as % of total fatty acids. Data are presented as mean ± SD (**A**) and mean (**B**,**C**), n = 3. The glycoglycerolipid composition and fatty acid composition, with mean ± SD, are reported in Tables S1 and S2, respectively.

Total lipid content ranged from 9% to 12% of Dry Cell Weight (DCW), being 12.4% ± 0.3 for ETS-05, 10.1% ± 1.2 for ETS-09 and 9.0% ± 0.7 for ETS-13 (Figure 1A). These outcomes are consistent with the lipid yields observed in various cyanobacterial species [37].

Lipid extracts were then analyzed by ERETIC ^1^H-NMR through integration of the diagnostic signals of the main glycoglycerolipids (GLs) [30] (Figure S5). The exact lipid class composition is reported in Table S1 with GLs being the main components, specifically featured by monogalactosyldiacylglycerols (MGDGs) (260–331.3 nmoles/mg lipid extract), digalactosyldiacylglycerols (DGDGs) (73.6–136.4 nmoles/mg lipid extract) and sulfoquinovosyldiacylglycerols (SQDGs) (87.6–162.3 nmoles/mg lipid extract) (Table S1). Figure 1B indicates the % of MGDG, DGDG and SQDG on the total GLs showing that the composition is quite similar among the different cyanobacterial strains with MGDG being the most abundant class, in line with the literature data [14].

Fatty acid composition was assessed by GCMS after transesterification of complex lipids in the corresponding Fatty Acid Methyl Ester (FAME) (Figure 1C). The exact FAME % is reported in Table S2 and the GCMS chromatograms are reported in Figure S6. All three species showed a predominance of Saturated Fatty Acids (SFAs), 16:0 being the most abundant molecule. ETS-05 profile revealed high quantity of PolyUnsaturated Fatty Acids, PUFAs (e.g., 18:4), which are completely absent in ETS-09 and less predominant in ETS-13 (18:2). In fact, these two latter species showed a predominance of MonoUnsaturated Fatty Acids, MUFAs (mainly 16:1 in ETS-09 and 18:1 in ETS-13), over PUFA. Data are consistent with the general concept of desaturation modification according to environmental temperature, where at the high temperatures, such the one used in this work, a general increase in MUFAs and SFAs has been observed at the expense of PUFAs [19,38]. Indeed, this phenomenon can be seen also when comparing the FAME composition of ETS-05 and ETS-09, grown at 30 and 35 °C, as described by Marcolongo et al. [15] and Moro et al. [17], respectively. ETS-13 has never been characterized for its FAs composition at other temperatures. However, comparing it with the one of the thermophilic cyanobacterium *Leptolyngbya* sp. grown at 30 °C, as described by Gara-Ali et al. [39], we can appreciate a similarity in SFAs amount and a reduction of 18:2.

### 3.3. Human Skin Fibroblasts Viability Assay

**Figure 2:**
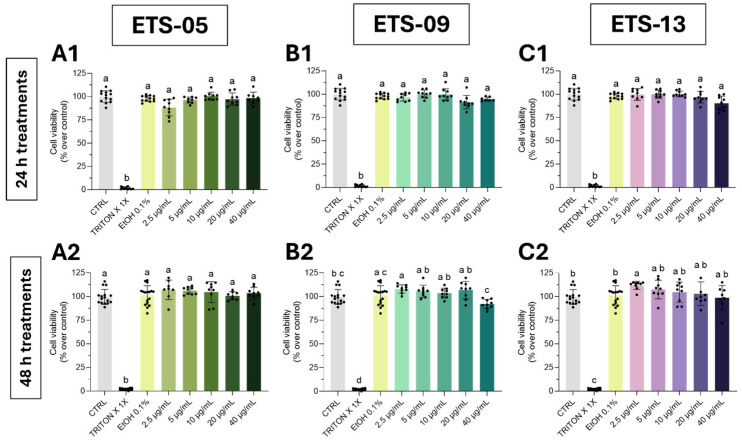
Cell viability analysis after BJ fibroblast exposure to total crude lipid extracts from ETS-05, ETS-09 (B1-B2), ETS-13 (C1-C2). (**A1**,**B1**,**C1**): cell viability after 24 h of treatment with total crude lipid extracts from ETS-05 (**A1**), ETS-09 (**B1**) and ETS-13 (**C1**). (**A2**,**B2**,**C2**): cell viability after 48 h of treatment with total crude lipid extracts from ETS-05 (**A2**), ETS-09 (**B2**) and ETS-13 (**C2**). After the incubation period, cell viability was calculated and data were compared to control values. Data are shown as the mean ± SD of three independent experiments, each carried out in triplicate. Statistical analysis was performed using GraphPad Prism 10 (Brown–Forsythe and Welch ANOVA tests followed by Dunnett’s multiple comparison test with individual variances computed for each comparison). Statistical significance was set at *p* < 0.05, and the results of the multiple comparisons are shown with letters (different letters show differences among data). The exact adjusted *p* values are listed in Table S3.

To analyze potential cellular toxicity, different concentrations of cyanobacterial total lipid extract (2.5–40 μg/mL), dissolved in ethanol, were used to treat human skin fibroblasts (BJ) for 24 and 48 h. Then, cell viability was estimated using the CCK-8 assay. Lipids did not show any cytotoxicity toward BJ fibroblasts with all the concentrations tested with a 24 h treatment (Figure 2A1,B1,C1). Instead, for longer exposure, although not significant, a small reduction in cell viability was determined, especially with the lipids extracted from ETS-09 and ETS-13 (Figure 2B2,C2).

### 3.4. Zebrafish Embryo/Larvae Developmental Toxicity Assay

**Figure 3:**
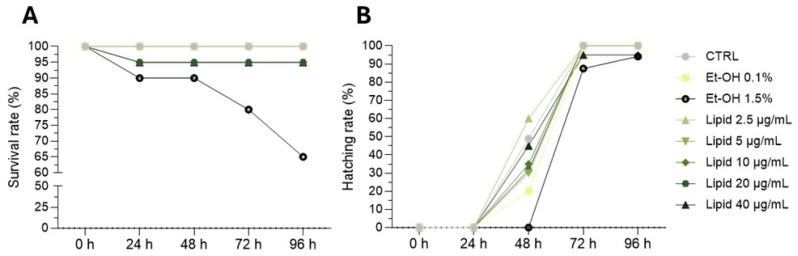
The effect of exposure to ETS-05 total crude lipid extract on the development of zebrafish embryos by FET test. Survival rates (**A**) and hatching rates (**B**) were counted at each time point. Et-OH 0.1% was used as a solvent control, and Et-OH 1.5% was used as a positive control to test reliability of the analysis.

Before the assessment of lipids anti-inflammatory and pro-resolution activity, an in vivo toxicological evaluation of *Phormidium* sp. ETS-05 total lipid extract was conducted according to the indication of the FET test (OECD Guideline No. 236) [33]. This test was conducted only with the lipids from this species given the higher yield produced by ETS-05 and the high amount of compound requested to perform this analysis. Different concentrations of lipids (2.5–40 μg/mL), dissolved in ethanol, were used for the treatments, starting at the 6-hpf stage and ending at 96-hpf with the media changed every 24 h. Since the mortality of the negative and positive control, calculated at 96 hpf, were of 0% and of 35%, respectively, the test achieved the FET acceptance criteria [33] (Figure 3A).

The survival rate after lipid exposure was slightly affected only by the higher concentration (20–40 μg/mL), indicating that, at these doses, lipids could disturb zebrafish development (Figure 3A). At all other concentrations, the absence of key negative morphological outcomes, as indicated by FET test—such as coagulation of embryos, lack of somite development, non-detachment of the tail-bud and absence of heartbeat—revealed that the tested molecules were not associated with toxicity or teratogenic effects. Also, the hatching rate was comparable to the negative and solvent controls (Figure 3B).

For the subsequent tests of anti-inflammatory capability, the highest concentration, 20–40 μg/mL, were discarded since they showed slight toxicity both in vitro and in vivo (Figure 2 and Figure 3, respectively).

### 3.5. Analysis of Anti-Inflammatory Capabilities on Zebrafish Larvae

The evaluation of the anti-inflammatory potential of the lipids extracted from the cyanobacterial strains studied in this work was performed with a zebrafish model of copper-induced inflammation, as described in [40]. Treatment with copper sulphate produces an oxidative stress and leads to a systemic inflammation that in turn generates an impairment of zebrafish development and a delay in the progression of processes like swim bladder insufflation and operculum bone ossification. Exposure of the larvae to bioactive molecules can contrast this lag in development [8,41], and this can be assessed by morphometric analyses of selected traits. This approach to investigate the anti-inflammatory potential of bioactive molecules was based on our previous works [8,41] in which we validated the anti-inflammatory and antioxidant potential of microbial polysaccharides using different experimental procedures, including the morphometric analysis of developmental delay.

Larvae were exposed to different concentrations of total crude lipid extracts and for different periods (24 and 48 h) to highlight any differences in the anti-inflammatory capabilities of lipids extracted from the different cyanobacterial species.

Representative images of 5-dpf zebrafish larvae after 48 h of exposure (CTRL, CuSO_4_ and CuSO_4_ + ETS-05 lipids) are described in Figure S3.

**Figure 4:**
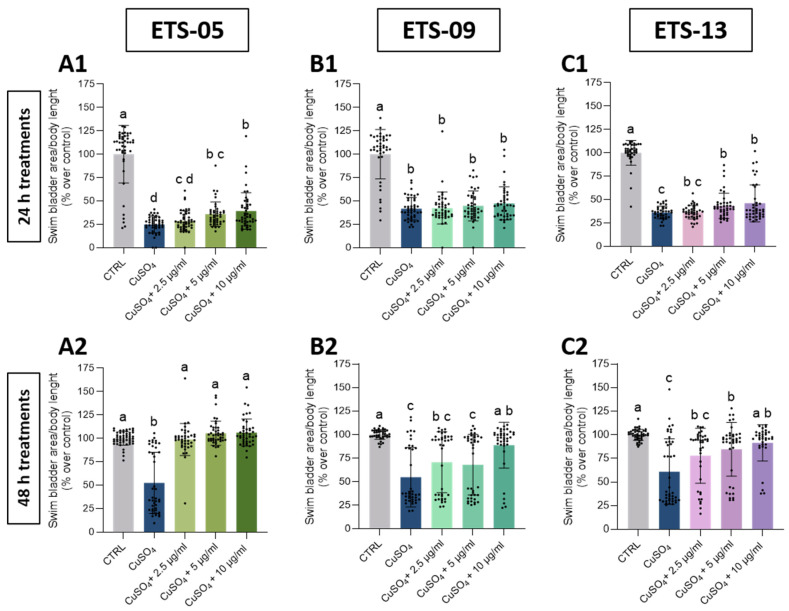
Recovery of developmentally appropriate size of swim bladder area after treatment with total crude lipid extracts. (**A1**,**B1**,**C1**): recovery from inflammation after 24 h of treatment with total crude lipid extracts from ETS-05 (**A1**), ETS-09 (**B1**) and ETS-13 (**C1**). (**A2**,**B2**,**C2**): recovery from inflammation after 48 h of treatment with total crude lipid extracts from ETS-05 (**A2**), ETS-09 (**B2**) and ETS-13 (**C2**). Data were compared to control values. The swim bladder area was normalized over body length and indicated as percentages over control. Black bars represent the mean ± SD of three independent experiments conducted with 15–20 larvae per treatment. Statistical analysis was performed using GraphPad Prism 10 (Brown–Forsythe and Welch ANOVA test followed by Dunnett’s T3 multiple comparisons test with individual variances computed for each comparison). Statistical significance was set at *p* < 0.05, and the results of the multiple comparisons are shown with letters (different letters show differences among data). The exact adjusted *p* values are listed in Table S4.

At the end of the treatments, swim bladder insufflation delay (Figure 4) was restored to WT parameters only with the lipids extracted from ETS-05. Specifically, the temporal scan of the treatments disclosed a progression in the morphological trait recovery, with a partial recovery of the swim bladder at 24 h (Figure 4A1,B1,C1) and a more substantial or complete recovery at 48 h for ETS-09, ETS-13 and ETS-05 lipids, respectively (Figure 4B2,C2,A2).

**Figure 5:**
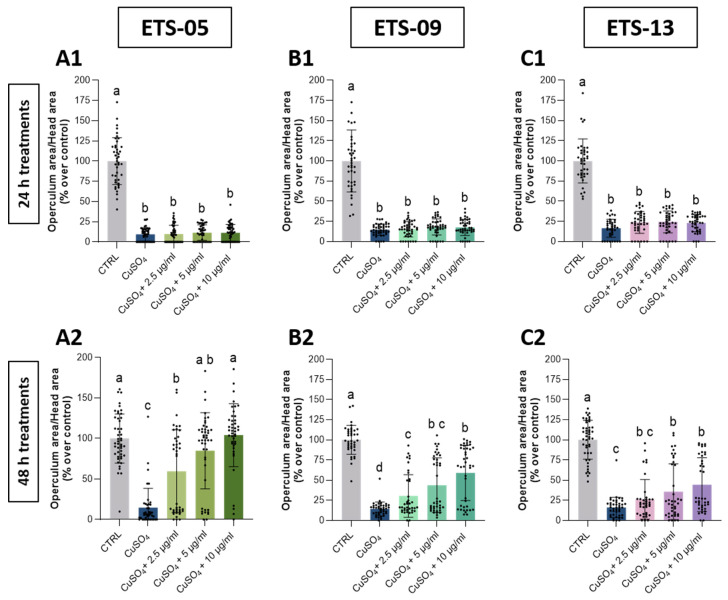
Recovery of developmentally appropriate bone area size after treatment with total crude lipid extracts. (**A1**,**B1**,**C1**): recovery from inflammation after 24 h of treatment with total crude lipid extracts from ETS-05 (**A1**), ETS-09 (**B1**) and ETS-13 (**C1**). (**A2**,**B2**,**C2**): recovery from inflammation after 48 h of treatment with total crude lipid extracts from ETS-05 (**A2**), ETS-09 (**B2**) and ETS-13 (**C2**). Data were compared to control values. The operculum bone area was normalized over head area and indicated as percentages over control. Black bars represent the mean ± SD of three independent experiments conducted with 15–20 larvae per treatment. Statistical analysis was performed using GraphPad Prism 10 (Brown–Forsythe and Welch ANOVA test followed by Dunnett’s T3 multiple comparisons test with individual variances computed for each comparison). Statistical significance was set at *p* < 0.05, and the results of the multiple comparisons are shown with letters (different letters show differences among data). The exact adjusted *p* values are listed in Table S5.

In contrast, the recovery of the ossification of the operculum was slower, with no rescue observed at 24 h and only a partial recovery detected after 48 h (Figure 5).

Interestingly, the operculum development was rescued to WT parameters only with the highest concentrations (5–10 μg/mL) of ETS-05 lipids, thus confirming that the total lipid extract of ETS-05 presents a stronger anti-inflammatory and antioxidant capability (Figure 5A2). Conversely, the inflamed siblings exposed only to FW after the copper sulphate treatment slightly recovered from the developmental delay. They exhibited only a minor progressive recovery from 24 to 48 h post-inflammation for swim bladder insufflation (Figure 4); the recovery was even more reduced for operculum ossification (Figure 5).

As reported by Zhang et al. [13], the bioactivity of GLs is associated with their chemical structures including the sugar moiety, the position of the glycerol linkage to the sugar, the length and location of the acyl chain and the anomeric configuration of the sugar. Moreover, the degree of saturation of the fatty acid chains of GLs also has an important role in the anti-inflammatory activity of this compound, as already shown by Bruno and collaborators, [9] on the activity of MGDG extracted from the target species ETS-05 grown at 30 °C.

Indeed, the differences in the anti-inflammatory activities of the different cyanobacterial extracts, both in extent and velocity of the recovery, could be due to the different degrees of fatty acid desaturation as well as to the presence of other minority bioactive compounds.

Moreover, it is interesting to notice that the overall trait recovery ability and the rate of recovery are lower than those identified with polysaccharides extracted from the target species ETS-05 [12] and from the therapeutic muds dominated by this species [41].

### 3.6. Swimming Behavior of Zebrafish Larvae After Lipid Treatments

**Figure 6:**
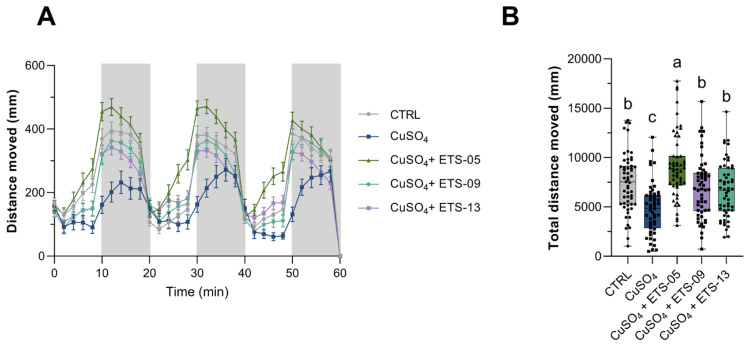
Analysis of locomotor behavior of zebrafish larvae after inflammation and treatment with lipid extracts. (**A**): recovery from inflammation after 48 h of treatment with lipid extracted from ETS-05, ETS-09 and ETS-13. (**B**): total distance moved by larvae depending on treatment. All experiments were performed using 10 μg/mL lipid extract. Behavioral analysis protocol: 10 min of light (white background) and 10 min of dark (grey background). Data was obtained considering 2 min intervals during the 60 min sessions. Black bars denote the mean ± SEM of three independent experiments conducted with 15–18 larvae per treatment in A and mean ± SD in B. Statistical analysis was performed using GraphPad Prism 9.5 (ordinary one-way ANOVA followed by Tukey’s multiple comparisons test). Statistical significance was set at *p* < 0.05, and the results of the multiple comparisons are shown with letters (different letters show differences among data). The exact adjusted *p* values are listed in Table S6.

In addition to the developmental delay, it is known that exposure to copper sulphate determines also a dysfunctional locomotor performance of zebrafish larvae [42,43] that can be analyzed using the zebrafish light–dark locomotion test. Here, this protocol was used to investigate the swimming impairment due to the inflammatory induction and the recovery obtained after treatment with the different 10 μg/mL lipid extracts for 48 h. The dark-to-light shift in this test usually leads to a quick drop in locomotor activity, while the light-to-dark change determines a rapid rise in motor behavior [44]. Even though we observed this regular trend in all samples, including inflamed larvae without lipid treatment, the latter displayed a reduction in locomotor activity, especially during the dark phases (Figure 6A).

In contrast to inflamed larvae, treatments with all the tested lipid extracts allowed the rescue of larvae movement, as shown in Figure 6A and confirmed by the calculation of the total distance moved (Figure 6B). Interestingly the recovery of the locomotor behavior was more pronounced with the lipids from ETS-05 and lower, but statistically significant, with the lipids from ETS-09 and ETS-13, as already shown by the morphological trait’s recovery. Moreover, as clearly shown by the tracks and the total distance moved (Figure 6A,B), the treatment with the lipids from ETS-05 are able not only to restore the locomotor impairment, with respect to the inflamed siblings, but also to improve the locomotor activity.

## 4. Conclusions

The study of cyanobacteria from thermal environments is of high interest as they produce a bunch of protective molecules that allow them to survive in extreme conditions and that can be exploited for therapeutic purposes. This study explored the production, composition and biological activity of lipids from the three most abundant thermophilic cyanobacterial strains isolated from the Euganean thermal muds: *Phormidium* sp. ETS-05, *Thermospirulina andreolii* ETS-09 and *Kovacikia euganea* ETS-13. The organisms were grown under optimal temperature conditions to assess the composition and bioactivity of their lipids. The growth temperatures were selected also considering the species-specific temperature inducing the highest growth in the mature muds of the Euganean Thermal District. The results showed that lipid content varied among strains, with ETS-05 exhibiting the highest lipid yield and the greatest abundance of PUFAs, particularly 18:4. In contrast, ETS-09 and ETS-13 primarily produced SFAs and MUFAs, reflecting their adaptation to higher temperature environments. No significant effects on human fibroblast viability were obtained by the treatment with different concentrations of ETS-05 lipids, thus demonstrating lack of in vitro cytotoxicity. Only a slight reduction in viability, even not statistically significant, was observed at higher concentrations during long-term exposure. Zebrafish embryonic toxicity assays further confirmed the safety of the lipids at concentrations up to 20 μg/mL.

Regarding the potential bioactivity of these molecules, all lipid extracts displayed anti-inflammatory effects in a zebrafish model of copper sulphate-induced inflammation. This was evidenced by the improved recovery of swim bladder insufflation and operculum bone ossification in treated larvae compared to the inflamed one. Particularly, ETS-05, besides the highest lipids production, determined the highest and fastest developmental recovery, a result further supported by the locomotor behavior recovery. The higher bioactive potential of the lipid of this strain could be linked to the FAs composition, confirming its target species status and emphasizing the importance of performing mud maturation at the suggested temperature range of 37–47 °C.

One limitation of this study is represented by the use of total lipid extracts without a previous fractionation into their components. It is known that even minor lipophilic components of Cyanobacteria, such as sterols, tocopherols and phospholipids, could have therapeutic proprieties [45,46]. For this reason, we cannot currently attribute the beneficial function we observed to a specific lipid subclass. However, in this work, differently from the past works in which the properties of specific classes of ETS-05 lipids have been studied [9,10,11], we decided to analyze the beneficial effect of total lipid extracts in order to better mimic the overall presence of this type of biomolecules in the therapeutic mud.

In conclusion, our study highlights the bioactivity of these cyanobacterial lipid extracts and underscores the importance of studying lipid production under growth conditions that replicate those experienced by the relative strains during Euganean mud maturation. This is critical since lipid composition, especially fatty acid desaturation, can be modified by temperature variations. Thus, to obtain bioactive molecules with a defined profile, the maturation temperature should be finely controlled.

Future studies will expand on these findings by assessing therapeutic potential in mammalian cell lines models. Moreover, studies on the possible synergistic beneficial effect of polysaccharides and lipids could be considered to broaden the knowledge of the therapeutic efficacy of Euganean peloids.

## Data Availability

The original contributions presented in this study are included in the article/Supplementary Materials. Further inquiries can be directed to the corresponding author(s).

## Supplementary Materials

The following supporting information can be downloaded at: https://www.mdpi.com/article/10.3390/biom15091301/s1, Figure S1: Schematic representation of the organization of the FET plates; Figure S2: Schematic representation of anti-inflammatory treatments performed on zebrafish larvae; Figure S3: Representation of the analysed morphological traits in zebrafish larvae; Figure S4: Representative picture of ETS-13 cultures; Figure S5: 1H-NMR spectra of total lipid extract of ETS-05, ETS-09 and ETS-13; Figure S6: GCMS chromatograms of Fatty Acid Methyl Ester (FAME); Table S1: Glycoglycerolipid composition; Table S2: Fatty Acid Methyl Ester (FAME) composition; Table S3: Exact adjusted *p* value resulted from Brown-Forsythe and Welch ANOVA test followed by post hoc Dunnett’s T3 multiple comparison test of Figure 2 (Analysis of BJ cells viability); Table S4: Exact adjusted *p* value resulted from Brown-Forsythe and Welch ANOVA test followed by post hoc Dunnett’s T3 multiple comparison test of Figure 3 (Analysis of swim bladder area); Table S5: Exact adjusted *p* value resulted from Brown-Forsythe and Welch ANOVA test followed by post hoc Dunnett’s T3 multiple comparison test of Figure 4 (Analysis of operculum bone ossification); Table S6: Exact adjusted *p* value resulted from ordinary one-way ANOVA followed by Tukey’s multiple comparisons test of Figure 6.
