# Supplementary material for: In Vivo Anti-Inflammatory Activity of Lipids Extracted from the Most Abundant Cyanobacterial Strains of the Therapeutic Euganean Thermal Muds
Source: Biomolecules. 2025 Sep 10;15(9):1301. doi: 10.3390/biom15091301 (PMC12467192; doi:10.3390/biom15091301)
Supplement: Supplementary file 1 [file biomolecules-15-01301-s001.zip › biomolecules-3822434-supplementary.pdf]

In vivo anti-inflammatory activity of lipids extracted from the most abundant cyanobacterial strains of the therapeutic Euganean thermal muds

Micol Caichiolo [1,2,†], Giuliana d'Ippolito [3], Angela Grazioso [3], Chiara Rampazzo [1], Angelica Marchetto [1], Fabrizio Caldara [2], Luisa Dalla Valle [1,*] and Nicoletta La Rocca [1,*]

Department of Biology, University of Padova, Via U. Bassi 58/b, 35131 Padova, Italy; micol.caichiolo@phd.unipd.it (M.C.); chiara.rampazzo.1@unipd.it (C.R.); marchetto.angelica.1998@gmail.com (A.M.)
2 Pietro D'Abano Thermal Studies Center, Via Jappelli 5, 35031 Padova, Italy; fabrizio.caldara@centrostuditermali.org
3 Institute of Biomolecular Chemistry, Centro Nazionale delle Ricerche, Via Campi Flegrei 34, 80078 Naples, Italy; giuliana.dippolito@cnr.it (G.d.); angela.grazioso@unina.it (A.G.)
*Correspondence: luisa.dallavalle@unipd.it (L.D.V.); nicoletta.larocca@unipd.it (N.L.R.); Tel.: +39-0498276188 (L.D.V.); +39-0498276273 (N.L.R.)

[†] Present address: Department of Comparative Biomedicine and Food Science, University of Padova, Via dell'Università 16, 35020 Legnaro, Italy.

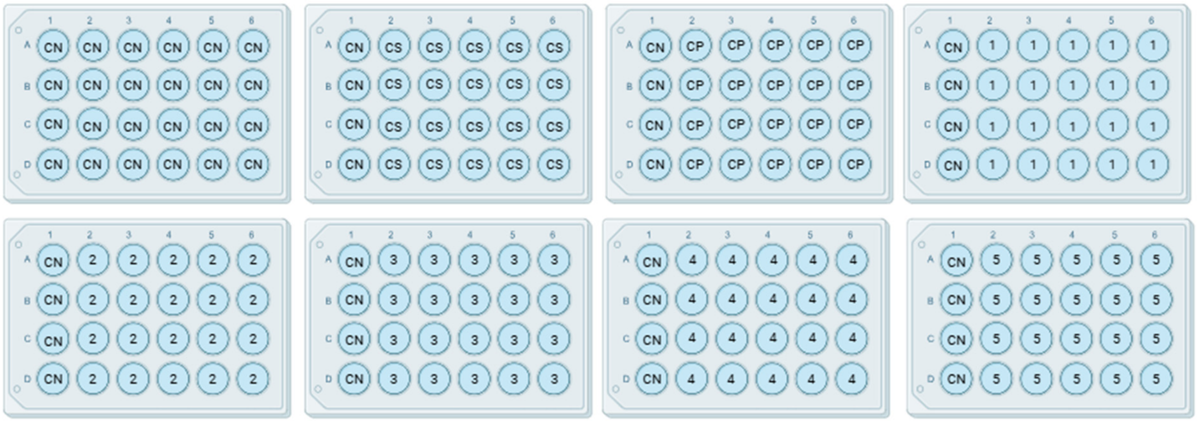

Figure S1. Schematic representation of the organization of the FET plates. CN: negative control (FW); CP: positive control (1.5% Et-OH); CS: solvent control (0.1% Et-OH); Lipid concentrations in Et-OH: 1= 40 μg/mL; 2 = 20 μg/mL; 3 = 10 μg/mL; 4 = 5 μg/mL; 5 = 2.5 μg/mL). Image created with Biorender.com.

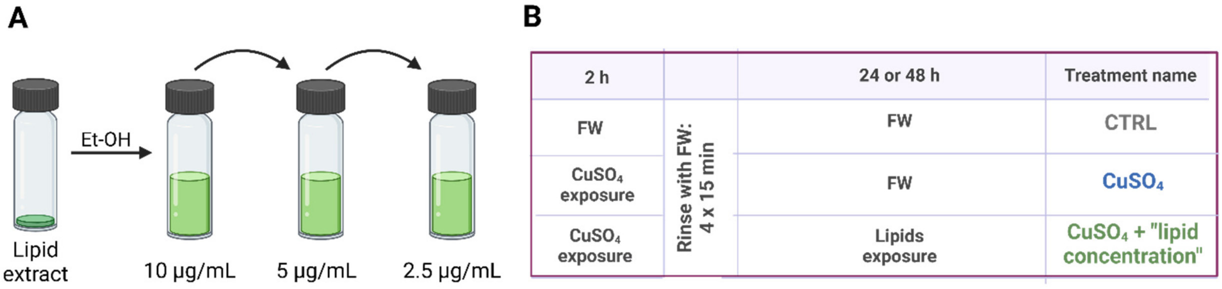

Figure S2. Schematic representation of anti-inflammatory treatments performed on zebrafish larvae. (A) Preparation of the lipid solutions used for the *in vivo* tests. (B) Scheme of the treatments performed for the morphological traits evaluation and the "Danio vision" experiment. The larvae were exposed to the three lipid solutions for 24- or 48-hours post-inflammation. The lipids from the different cyanobacterial strains were tested separately. The lipids from the different cyanobacterial strains were tested in parallel in the same experiment. Image created with Biorender.com.

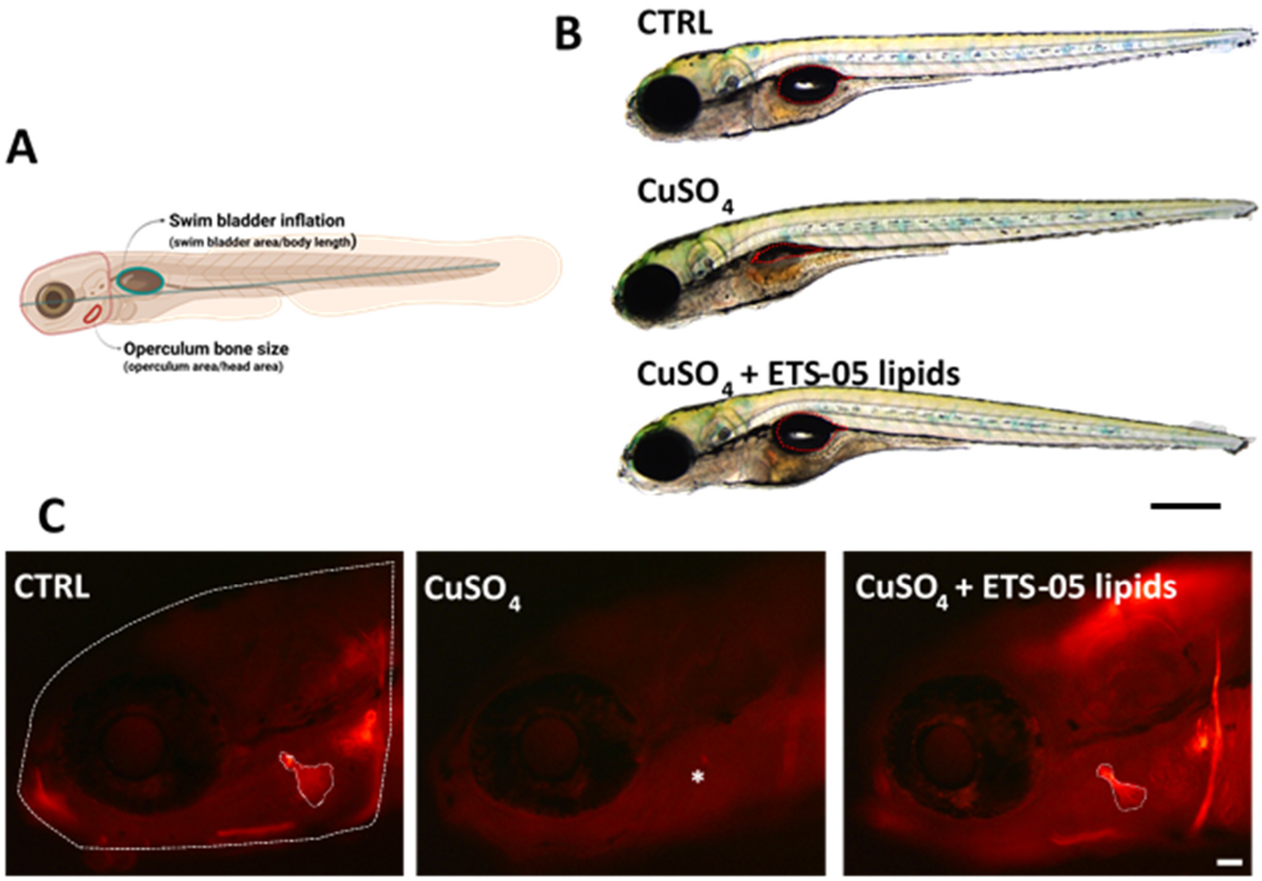

Figure S3. Representation of the analysed morphological traits in zebrafish larvae:area of the swim bladder, body length (used for the normalization), operculum bone area and head area (used for the normalization) (A) Scheme representing the morphometric traits analysed on zebrafish larvae (Image created with Biorender.com). (B) Micrographs showing reduced inflation of swim bladder due to $CuSO_4 \cdot 5H_2O$ exposure ($CuSO_4$) and rescue of the inflation after 48 h ETS-05 5 µg/mL lipid treatment ($CuSO_4$ + ETS-05 lipids). Sibling larvae (CTRL) are shown for comparison. Swim bladder is highlighted by red dotted lines. Scale bar = 500 µm. (C) Picture of the head of 5-dpf, stained with alizarin red S and showing the head and the operculum area evaluated though morphometric analyses. The operculum is highlighted by white dotted lines or white star. The micrographs show reduced operculum ossification after $CuSO_4 \cdot 5H_2O$ exposure ($CuSO_4$) and ossification rescue due to 48 h ETS-05 5µg/mL lipids treatment ($CuSO_4$ + ETS-05 lipids). Sibling larvae (CTRL) are shown for comparison. Scale bar = 50 µm.

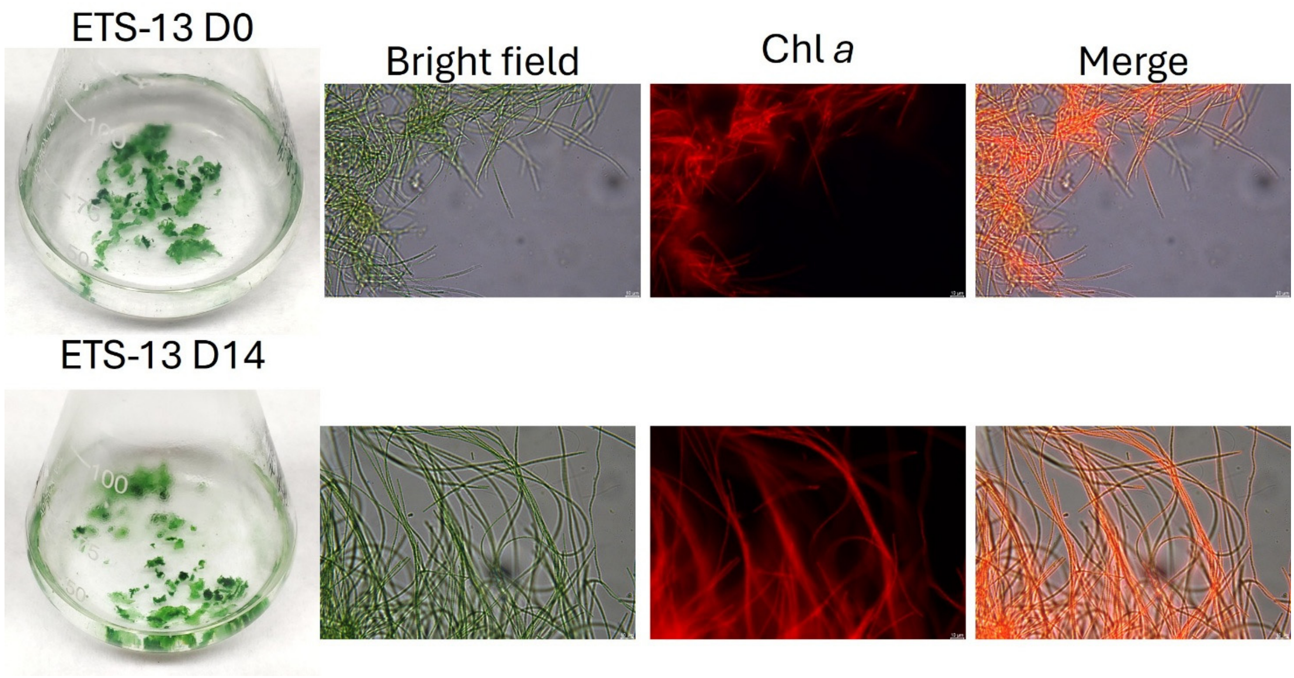

Figure S4. Representative picture of ETS-13 cultures at the beginning and at the end of the growth (on the left) and optical microscopy images of ETS-13 cells (on the right). Images were acquired in brightfield and using the autofluorescence of chlorophyll *a* (Chl *a*) to check the viability of the cells. Bar scale: 10 µm. D0 and D14 indicate Day 0 and Day 14 of the growth, respectively.

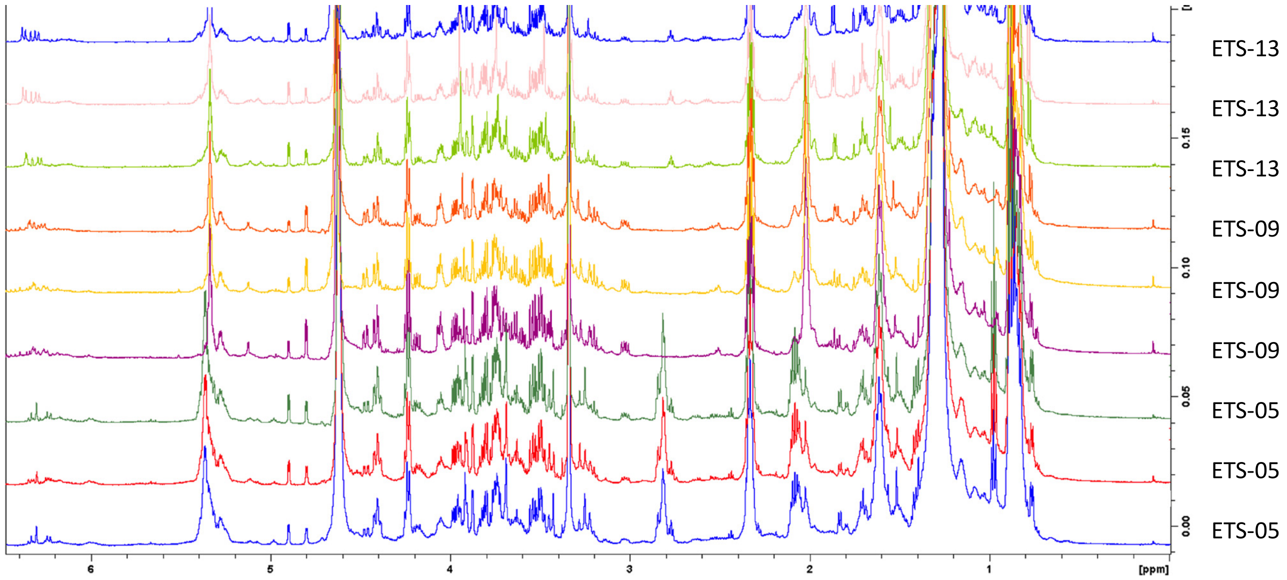

Figure S5. ^{1}H-NMR spectra of total lipid extract of ETS-05, ETS-09 and ETS-13. Data reported are the results of three replicated for each species.

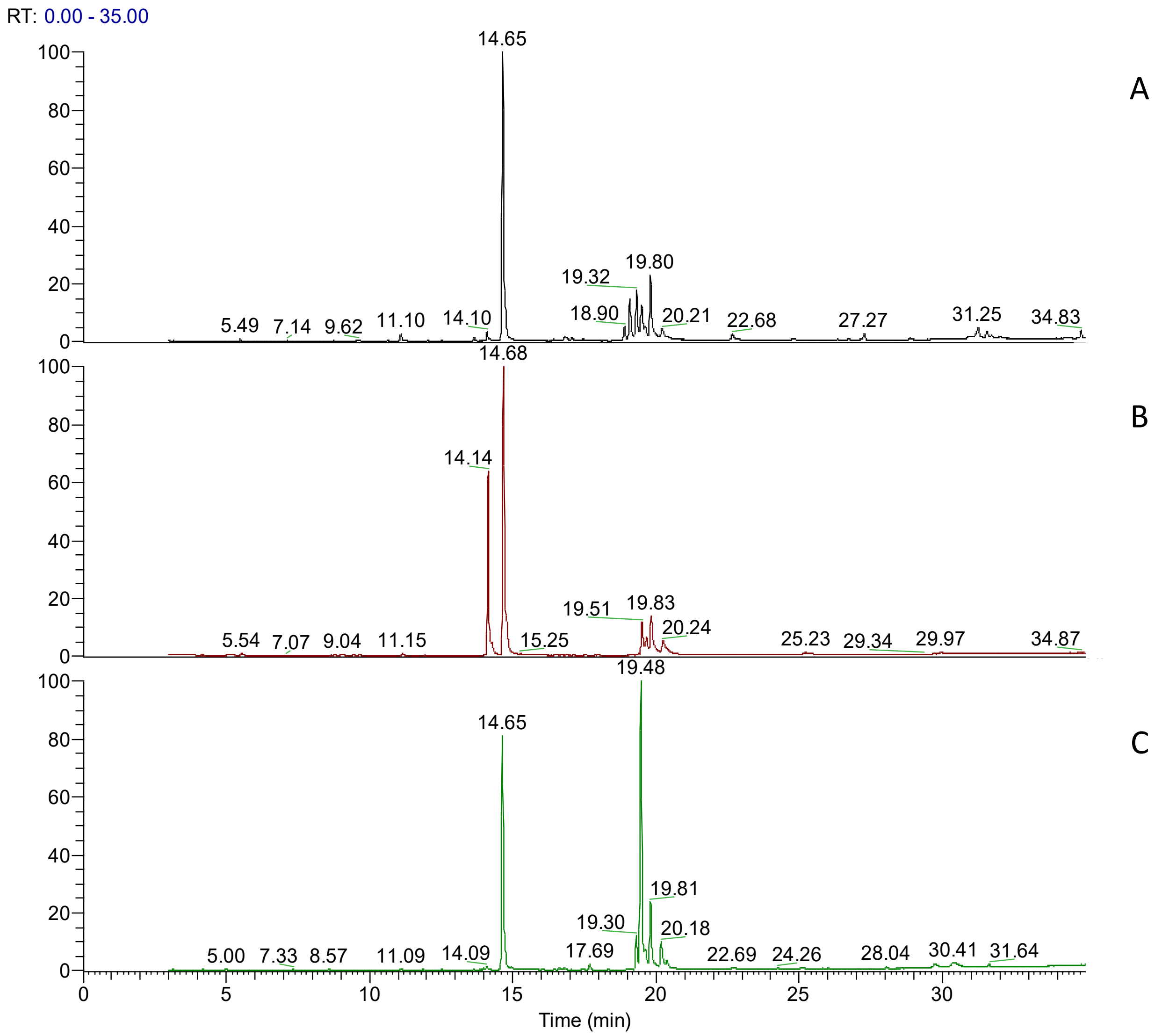

Figure S6. GCMS chromatograms of Fatty Acid Methyl Ester (FAME) of ETS-05 (A), ETS-09 (B) and ETS-13 (C).

Table S1. Glycoglycerolipid composition (nmol/mg lipid extract ± SD) of ETS-05, ETS-09 and ETS-13. n =3.

LIPID CLASS	ETS-05	ETS-09	ETS-13
MGDG	260.1 ± 49.1	331.3 ± 93.1	332.9 ± 30.5
DGDG	97.9 ± 0.8	73.6 ± 7.9	136.4 ± 10.8
SQDG	87.6 ± 5.2	162.3 ± 11.6	122.0 ± 18.1

Table S2. Fatty Acid Methyl Ester (FAME) composition (% ± SD) of ETS-05, ETS-09 and ETS-13. n =3.

FAME	ETS-05	ETS-09	ETS-13
16:1 ω7	1.7 ± 0.18	30.5 ± 1.03	
16:0	61.7 ± 4.47	56.1 ± 0.96	45.5 ± 1.01
18:4 ω1	13.9 ± 3,46		
18:2 ω6	9.3 ± 0.41		4.6 ± 1.02

18:1 ω9	10.4 ± 1.50	6.1 ± 0.34	46.5 ± 2.02
18:1	0.0	3.0 ± 0.93	
18:0	3.0 ± 1.00	4.3 ± 0.62	3.3 ± 0.28

Table S3. Exact adjusted P value resulted from Brown-Forsythe and Welch ANOVA test followed by post hoc Dunnett's T3 multiple comparison test of Figure 2 (Analysis of BJ cells viability).

**Dunnett's T3 multiple comparisons test**	**Adjusted P Value A1**	**Adjusted P Value A2**	**Adjusted P Value B1**	**Adjusted P Value B2**	**Adjusted P Value C1**	**Adjusted P Value C2**
CTRL vs. TRITON X 1X	<0,0001	<0,0001	<0,0001	<0,0001	<0,0001	<0,0001
CTRL vs. EtOH 0.1%	0,9989	>0,9999	0,9989	>0,9999	0,9989	>0,9999
CTRL vs. 2.5 μg/mL	0,0875	0,8173	0,9571	0,0479	>0,9999	0,0009
CTRL vs. 5 μg/mL	0,8644	0,2089	>0,9999	0,6506	>0,9999	0,6590
CTRL vs. 10 μg/mL	>0,9999	0,9915	>0,9999	0,9525	>0,9999	0,9878
CTRL vs. 20 μg/mL	0,9998	>0,9999	0,2167	0,7061	0,9960	>0,9999
CTRL vs. 40 μg/mL	>0,9999	0,9831	0,2922	0,1726	0,1320	>0,9999
TRITON X 1X vs. EtOH 0.1%	<0,0001	<0,0001	<0,0001	<0,0001	<0,0001	<0,0001
TRITON X 1X vs. 2.5 μg/mL	<0,0001	<0,0001	<0,0001	<0,0001	<0,0001	<0,0001
TRITON X 1X vs. 5 μg/mL	<0,0001	<0,0001	<0,0001	<0,0001	<0,0001	<0,0001
TRITON X 1X vs. 10 μg/mL	<0,0001	<0,0001	<0,0001	<0,0001	<0,0001	<0,0001
TRITON X 1X vs. 20 μg/mL	<0,0001	<0,0001	<0,0001	<0,0001	<0,0001	<0,0001
TRITON X 1X vs. 40 μg/mL	<0,0001	<0,0001	<0,0001	<0,0001	<0,0001	<0,0001
EtOH 0.1% vs. 2.5 μg/mL	0,1922	0,9853	>0,9999	0,4420	0,9999	0,0254
EtOH 0.1% vs. 5 μg/mL	0,9985	0,8677	0,9665	0,9891	0,9880	0,9531
EtOH 0.1% vs. 10 μg/mL	0,9428	>0,9999	>0,9999	>0,9999	0,7935	>0,9999

EtOH 0.1% vs. 20 μg/mL	>0,9999	>0,9999	0,4181	0,9758	>0,9999	>0,9999
EtOH 0.1% vs. 40 μg/mL	>0,9999	>0,9999	0,3486	0,1042	0,2675	>0,9999
2.5 μg/mL vs. 5 μg/mL	0,3944	>0,9999	0,8056	0,9998	>0,9999	0,9884
2.5 μg/mL vs. 10 μg/mL	0,0606	>0,9999	0,9919	0,7271	>0,9999	0,7784
2.5 μg/mL vs. 20 μg/mL	0,4008	0,9120	0,7821	>0,9999	0,9991	0,6942
2.5 μg/mL vs. 40 μg/mL	0,2756	>0,9999	0,9949	<0,0001	0,1990	0,3342
5 μg/mL vs. 10 μg/mL	0,5434	>0,9999	>0,9999	>0,9999	>0,9999	>0,9999
5 μg/mL vs. 20 μg/mL	>0,9999	0,2895	0,1463	>0,9999	0,9856	>0,9999
5 μg/mL vs. 40 μg/mL	>0,9999	0,9979	0,1093	0,0038	0,1032	0,9390
10 μg/mL vs. 20 μg/mL	0,9954	0,9994	0,3341	0,9998	0,9215	>0,9999
10 μg/mL vs. 40 μg/mL	>0,9999	>0,9999	0,5808	0,0049	0,0671	0,9993
20 μg/mL vs. 40 μg/mL	>0,9999	0,9991	0,9798	0,0244	0,7158	>0,9999

Table S4. Exact adjusted P value resulted from Brown-Forsythe and Welch ANOVA test followed by post hoc Dunnett's T3 multiple comparison test of Figure 3 (Analysis of swim bladder area).

**Dunnett's T3 multiple comparisons test**	**Adjusted P Value A1**	**Adjusted P Value A2**	**Adjusted P Value B1**	**Adjusted P Value B2**	**Adjusted P Value C1**	**Adjusted P Value C2**
CTRL vs. $CuSO_4$	<0,0001	<0,0001	<0,0001	<0,0001	<0,0001	<0,0001
CTRL vs. $CuSO_4$+ 2,5 μg/ml	<0,0001	>0,9999	<0,0001	<0,0001	<0,0001	0,0004
CTRL vs. $CuSO_4$ + 5 μg/ml	<0,0001	0,1570	<0,0001	<0,0001	<0,0001	0,0168
CTRL vs. $CuSO_4$ + 10 μg/ml	<0,0001	0,1402	<0,0001	0,0548	<0,0001	0,1417
$CuSO_4$ vs. $CuSO_4$ + 2,5 μg/ml	0,4732	<0,0001	>0,9999	0,2760	>0,9999	0,1876
$CuSO_4$ vs. $CuSO_4$ + 5 μg/ml	0,0002	<0,0001	0,9853	0,4927	0,0368	0,0118
$CuSO_4$ vs. $CuSO_4$ + 10 μg/ml	0,0002	<0,0001	0,5906	<0,0001	0,0449	<0,0001
$CuSO_4$+ 2,5 μg/ml vs. $CuSO_4$ + 5 μg/ml	0,0634	0,3817	0,9995	>0,9999	0,0548	0,9695
$CuSO_4$+ 2,5 μg/ml vs. $CuSO_4$ + 10 μg/ml	0,0189	0,3121	0,8673	0,0675	0,0580	0,1838

$CuSO_4$ + 5 µg/ml vs. $CuSO_4$ + 10 µg/ml	0,9787	>0,9999	0,9971	0,0183	0,9976	0,9096

Table S5. Exact adjusted P value resulted from Brown-Forsythe and Welch ANOVA test followed by post hoc Dunnett's T3 multiple comparison test of Figure 4 (Analysis of operculum bone ossification).

Dunnett's T3 multiple comparisons test	Adjusted P Value A1	Adjusted P Value A2	Adjusted P Value B1	Adjusted P Value B2	Adjusted P Value C1	Adjusted P Value C2
CTRL vs. $CuSO_4$	<0,0001	<0,0001	<0,0001	<0,0001	<0,0001	<0,0001
CTRL vs. $CuSO_4$+ 2,5 µg/ml	<0,0001	0,0005	<0,0001	<0,0001	<0,0001	<0,0001
CTRL vs. $CuSO_4$ + 5 µg/ml	<0,0001	0,5152	<0,0001	<0,0001	<0,0001	<0,0001
CTRL vs. $CuSO_4$ + 10 µg/ml	<0,0001	0,9997	<0,0001	<0,0001	<0,0001	<0,0001
$CuSO_4$ vs. $CuSO_4$ + 2,5 µg/ml	>0,9999	<0,0001	>0,9999	0,0081	0,1239	0,2580
$CuSO_4$ vs. $CuSO_4$ + 5 µg/ml	0,9480	<0,0001	0,4841	<0,0001	0,0650	0,0160
$CuSO_4$ vs. $CuSO_4$ + 10 µg/ml	0,9822	<0,0001	0,7139	<0,0001	0,1228	<0,0001
$CuSO_4$+ 2,5 µg/ml vs. $CuSO_4$ + 5 µg/ml	0,9974	0,1830	0,8577	0,4075	>0,9999	0,8361
$CuSO_4$+ 2,5 µg/ml vs. $CuSO_4$ + 10 µg/ml	0,9996	0,0003	0,9623	0,0008	>0,9999	0,0785
$CuSO_4$ + 5 µg/ml vs. $CuSO_4$ + 10 µg/ml	>0,9999	0,3160	>0,9999	0,3707	0,9998	0,9433

Table S6. Exact adjusted P value resulted from ordinary one-way ANOVA followed by Tukey's multiple comparisons test of Figure 6.

Tukey's multiple comparisons test	Adjusted P Value
CTRL vs. $CuSO_4$	0,0001
CTRL vs. $CuSO_4$ + ETS-05	0,0293
CTRL vs. $CuSO_4$+ ETS-09	0,8879
CTRL vs. $CuSO_4$+ ETS-13	0,6719
$CuSO_4$ vs. $CuSO_4$ + ETS-05	<0,0001
$CuSO_4$ vs. CuSO4 + ETS-09	0,0040
$CuSO_4$ vs. $CuSO_4$ + ETS-13	0,0150

$CuSO_4$ + ETS-05 vs. $CuSO_4$ + ETS-09	0,0014
$CuSO_4$ + ETS-05 vs. $CuSO_4$+ ETS-13	0,0003
$CuSO_4$ + ETS-09 vs. $CuSO_4$ + ETS-13	0,9942
